# Early stop of progesterone supplementation after confirmation of pregnancy in IVF/ICSI fresh embryo transfer cycles of poor responders does not affect pregnancy outcome

**DOI:** 10.1371/journal.pone.0201824

**Published:** 2018-08-09

**Authors:** Song-Po Pan, Kuang-Han Chao, Chu-Chun Huang, Ming-Yih Wu, Mei-Jou Chen, Chin-Hao Chang, Jehn-Hsiahn Yang, Yu-Shih Yang, Shee-Uan Chen

**Affiliations:** 1 Department of Obstetrics and Gynecology, National Taiwan University Hospital, Taipei, Taiwan; 2 Livia Shanyu Wan Scholar, College of Medicine, National Taiwan University; 3 Department of Medical Research, National Taiwan University Hospital, Taipei, Taiwan; University of Crete, GREECE

## Abstract

Previous studies indicated that progesterone can be withdrawn at the time of the first positive β-hCG test without compromising the clinical pregnancy outcome in normal ovarian responder. However, the effect of early stop of progesterone supplementation for patients with poor ovarian response (POR) has not been investigated. This study retrospectively collected data from patients with POR in 156 IVF/ICSI fresh embryo transfer (ET) cycles in single tertiary center from January 2010 to June 2016. All the patients met ESHRE consensus, the Bologna criteria, of POR and had hCG injection for luteal phase support (LPS) on day 2, 5 and 8 after ovum pick-up. The pregnant patients were divided into two groups: early stop group represented those who stopped LPS from day of positive pregnancy test; control group represented those who kept progesterone supplementation till gestational age of 9 weeks. There were no significant differences in age, BMI, parity, hormone data, number of follicles>10(mm), endometrial thickness and number of embryos transferred between the two groups. After adjustment for possible confounders with multivariate logistic regression analysis, the clinical pregnancy rates (55.0% vs. 57.1%, P = 0.35), ongoing pregnancy rates (47.0% vs. 46.4%, P = 0.66), miscarriage rates (34.0% vs. 26.7%, P = 0.66) and live-birth rates (44.0% vs. 46.4%, P = 0.41) were not statistically different between early stop group and the control group. Our study indicates that early stop of progesterone supplementation on the day of positive pregnancy test for patients of POR using hCG as LPS in fresh ET cycles does not affect pregnancy outcome.

## Introduction

In a natural reproductive cycle, a luteinizing hormone (LH) surge triggers ovulation of pre-ovulatory follicle and formation of corpus luteum (CL). And then, pulsatile secretion of LH maintains CL function that produces progesterone and estradiol. The progesterone induces secretory transformation of uterine endometrium [1] and facilitates receptivity of endometrium and implantation of embryo [2]. After implantation, embryonic human chorionic gonadotropin (hCG) rescues the CL through induction of anti-apoptotic protein Mcl-1 and maintains its life span to around 9 weeks of gestation [3]. The CL continues to secrete estrogen and progesterone for maintenance of pregnancy. After luteal-placental shift, the placenta produces sufficient steroid hormones and replaces the function of CL. If no pregnancy occurs, the CL gradually undergoes apoptosis with its life span for only 14 days, and then the menstruation will come.

In the cycle of controlled ovarian stimulation (COS) for in vitro fertilization (IVF) and embryo transfer (ET), after administration of hCG for triggering of final oocyte maturation, supraphysiological levels of progesterone and estrogen are secreted by multiple CL. The high serum concentration of steroid hormones may result in profound negative feedback to inhibit the LH release that may lead to earlier luteolysis [4–6]. In addition, the uses of gonadotropin-releasing hormone (GnRH) agonists or antagonists in the follicular phase may lead to various degrees of LH suppression in the luteal phase. It’s well-documented that progesterone supplementation is important during the time between the disappearance of exogenous hCG administered for simulating LH surge and the rise in endogenous hCG during early implantation [7]. Thus, if without good quality of LPS, compromised pregnancy rates may occur in COS cycles because of luteal phase defects [8,9]. LPS has been demonstrated to be beneficial for pregnancy outcome by improving the clinical pregnancy rate and live birth rate of IVF-ET cycles [8,9].

For the reasons above, LPS with progesterone, estrogen, hCG or GnRH agonist have been used in IVF -ET cycles, although the ideal method has not been clearly determined [9–15]. The hCG has been concerned for its increasing risk of ovarian hyperstimulation syndrome (OHSS). In addition, there is still no international consensus about the optimal duration of LPS [16]. Some investigators indicated that progesterone can be withdrawn at the time of the first positive β-HCG test without compromising the clinical pregnancy rate or live birth rate [7,12,17,18]. But, some doctors discontinued progesterone supplementation at 7 weeks of pregnancy and the other doctors continued it until the 12 weeks [19–22]. It was thought to be better to continue progesterone than to take a risk of miscarriage using the earlier stop [19–21]. However, the effect of early stop of progesterone supplementation or continuation for patients with poor ovarian response (POR) has not been investigated.

While the use of hCG for LPS is effective, it is associated with an increased risk of OHSS [23]. The release of vasoactive substances secreted by the ovaries under hCG stimulation may play a key role in triggering this syndrome [24]. Restoring a timely hCG signal during the window of implantation has been shown to enhance CL rescue and secretion of steroid hormones [25]. In this study, we have focused on patients of POR using hCG for LPS, who are considered to be the lower risk of being OHSS. We attempted to explore whether early stop of progesterone supplementation after confirmation of pregnancy in fresh ET cycles of patients with POR would affect the reproductive outcome or not.

## Materials and methods

### Study population

In this study, the records of patients who had POR administered with hCG for LPS in IVF/ICSI with fresh ET cycles from January 2010 to June 2016 were reviewed. POR was in accordance with ESHRE consensus on the definition of poor response to ovarian stimulation for IVF with the Bologna criteria [26]. At least two of the following three features must be present: (i) Advanced maternal age (≥40 years) or any other risk factor for POR; (ii) A previous POR (≤3 oocytes with a conventional stimulation protocol); (iii) An abnormal ovarian reserve test (i.e. AFC, 5–7 follicles, AMH, 0.5–1.1 ng/ml or basal FSH > 10–15 mIU/mL). Patients who were non-pregnant, pregnant with serum level hCG < 20 IU/L and lost follow-up were excluded. The inclusive cycles were divided into two groups: early stop group who stopped LPS from day of positive pregnancy test, control group who kept progesterone supplementation till 9 weeks. This study was approved by the Institutional Review Board of our hospital.

### Treatment protocol and oocyte retrieval

All patients were treated with standard gonadotropin releasing hormone (GnRH) agonist or antagonist protocols as previously described [27,28]. Follicles were stimulated with use of a combination of one of FSH (Gonal-F; Merck-Serono, Rome, Italy; Puregon, MSD, Ravensburg, Germany; Elonva, corifollitropin alfa, MSD) and hMG (Menopur; Ferring Pharmaceuticals, Saint-Prex, Switzerland), with doses determined by ovarian response as measured by serum estradiol levels and ultrasound evaluation. A 6500 IU dose of hCG, (Ovidrel, Merck-Serono) was administered when ≥ 2 leading follicles reached 18 mm in diameter with compatible estradiol level. ovum pick-up (OPU) was scheduled 34–36 hours later.

### Fertilization, embryo grading and embryo transfer

Fertilization was performed with IVF or ICSI, according to individual condition of total sperm counts, motility and morphology in 39–41 hours after hCG administration. All the embryos were classified according to the shape of the blastomeres and the amount of detached nuclear fragments. Embryo transfer was performed from 2 to 3 days after OPU. A maximum of four embryos were transferred back into the uterus.

### Luteal phase support

All the patient received three doses of hCG, 1500 IU (Pregnyl®; N.V. Organon, Oss, Holland) on day 2, 5, and 8 after OPU plus 90 mg vaginal gel (Crinone®; progesterone; Fleet Laboratories Limited., Watford, Hertfordshire, UK) one tube daily started on day 2 after OPU. Serum β-hCG and P levels were measured on day 16 after OPU, and β-hCG level ≥ 20 mIU/mL was considered as pregnant. LPS was discontinued if the β-HCG was negative. The early stop or continuation of progesterone supplementation after confirmation of pregnancy was dependent on different doctor’s ideas. This decision can be treated as a random process in the selections of early stop group and control group. We retrospectively divided the pregnant patients into two groups: one stopped LPS right after the confirmation of pregnancy and the other had their LPS cessation after 9 weeks of gestation.

### Hormone assessment

Serum samples were analyzed using Immulite 2000 reproductive hormone assays (Diagnostic Product Corporation, Siemens, Los Angeles, CA, USA). The sensitivity was 0.1 mIu/ml for FSH; 0.05 mIu/ml for LH; 15 pg/ml for estradiol and 0.1 ng/ml for P. Intra-assay and inter-assay coefficients of variation were, 3.6% and, 4.3% for FSH;, 4.8% and, 10.7% for LH;, 6.7% and, 9.7% for estradiol and, 9.7% and, 12.2% for progesterone, respectively.

### Follow-up of pregnancy

A serum β-hCG pregnancy test was performed 16 days after OPU. The β-hCG measurement was repeated within the first 7 days after the first one, to rule out biochemical pregnancy. Clinical pregnancy with fetal heart activity was ascertained by transvaginal sonography at 7 weeks of gestation, respectively. The ongoing pregnancy was defined as continuation for at least 12 weeks of gestational age. Live births were followed for all of cases.

### Early stop of LPS

On the day of the pregnancy test, once positive serum level of β-HCG was confirmed, patients stop LPS immediately at the same day or continue progesterone supplementation to 9 weeks of gestation, according to different doctor’s principles. For both groups, all pregnancies were followed to delivery or other obstetric outcomes.

### Statistical analysis

The continuous data of demographic and clinical characteristics of patients were summarized as mean ± standard deviations (SD) with range (minimum, maximum). The categorical data were expressed as number percentage. The demographic and clinical data of the control group and the study group were compared with independent t-test for continuous data or Chi-square test for categorical data. Multivariate logistic regression analyses were performed to evaluate the association of pregnancy outcomes with early stop of progesterone supplement or not, while controlling for some confounders. Adjusted odds ratio was calculated. All tests were two-tailed, and a value of *P* < 0.05 was considered to indicate statistical significance with a confidence level of 95%. Data analysis was performed with SAS statistical software version 9.4 for Windows (SAS Institute Inc., Cary, NC, USA).

## Results

**Fig 1:**
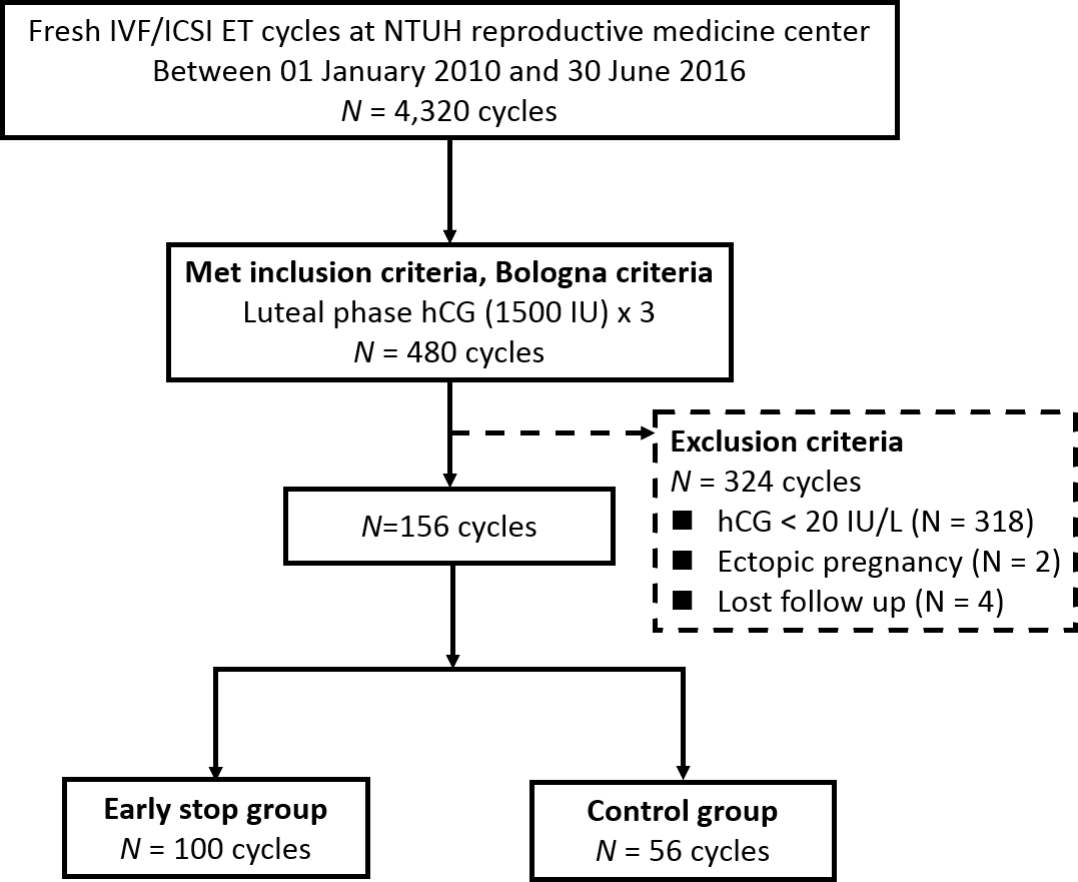
Flow chart of study procedures. From eligible 4,320 cycles with the initial criteria of fresh ET, 480 cycles met the Bologna criteria of POR. Finally, 156 cycles were selected after screening with exclusion criteria. At every follow-up visit, regular evaluations and assessments were made in both groups.Totally, 156 cycles with POR received fresh ET using LPS consisting of three doses of hCG (1500IU) plus Crinone 90 mg qd beginning 2 days after OPU were included in this study. One hundred cycles were in the study group with early stop of progesterone and 56 were in the control group without early stop. There were no statistically significant differences observed in baseline characteristics between two groups, as age (39.5 ± 3.8 vs. 39.9 ± 3.1 year; P = 0.51), body mass index (BMI) (21.8 ± 2.5 vs. 22.3 ± 2.8 kg/m2; P = 0.34), causes of infertility (P = 0.91), gravida (0.91 ± 1.1.5 vs. 0.96 ± 0.94; P = 0.77), parity (0.25 ± 0.55 vs. 0.16 ± 0.41; P = 0.26), previous artificial abortion (0.14 ± 0.37 vs. 0.21 ± 0.49; P = 0.33), spontaneous abortion (0.48 ± 0.79 vs. 0.52 ± 0.71; P = 0.77) and ectopic pregnancy (0.05 ± 0.26 vs. 0.04 ± 0.19; P = 0.69). The general demographics of the patient cohort are listed below (Table 1).

From eligible 4,320 cycles with the initial criteria of fresh ET, 480 cycles met the Bologna criteria of POR. Finally, 156 cycles were selected after screening with exclusion criteria. At every follow-up visit, regular evaluations and assessments were made in both groups and the flow chart of study procedures is shown in Fig 1.

**Table 1:** Demographics of patients.

	Early stop group (N = 100)		Control group (N = 56)		P-value
Age (years)	39.5 ± 3.8	(28–46)	39.9 ± 3.1	(31–45)	0.51 ^NS^
BMI (kg/m^2^)	21.8 ± 2.5	(16.8–29.3)	22.3 ± 2.8	(18.2–31.6)	0.34 ^NS^
Causes of infertility					0.91 ^NS^
Age	52	(52%)	29	(51.8%)	
Tubal	16	(16%)	9	(16.2%)	
Male	7	(7%)	3	(5.3%)	
Ovarian	12	(12%)	10	(17.8%)	
Uterine	8	(8%)	3	(5.3%)	
Endometriosis	5	(5%)	2	(3.6%)	
Gravida	0.91 ± 1.15	(0–6)	0.96 ± 0.94	(0–4)	0.77 ^NS^
Parity	0.25 ± 0.55	(0–3)	0.16 ± 0.41	(0–2)	0.26 ^NS^
Previous abortion					
Artificial abortion	0.14 ± 0.37	(0–2)	0.21 ± 0.49	(0–2)	0.33 ^NS^
Spontaneous abortion	0.48 ± 0.79	(0–4)	0.52 ± 0.71	(0–3)	0.77 ^NS^
Previous ectopic pregnancy	0.05 ± 0.26	(0–2)	0.04 ± 0.19	(0–1)	0.69 ^NS^

Data are means ± SD for continuous variables and number (%) for categorical variables. P-value from χ2 test as appropriate.

^NS^: not statistically significant.

The proportion of individual protocol in each group has no difference (P = 0.15). There were no statistically significant differences observed in serum levels of baseline FSH (12.8 ± 3.8 vs. 13.7 ± 6.4 mIU/mL; P = 0.36), LH (5.0 ± 1.8 vs. 5.7 ± 4.3 mIU/mL; P = 0.23), estradiol (36.2 ± 17.6 vs. 34.8 ± 13.8 pg/mL; P = 0.60), estradiol on the hCG injection day (797.7 ± 475.2 vs. 953.5 ± 530.8 pg/mL; P = 0.06), progesterone on hCG injection day (0.5 ± 0.6 vs. 0.6 ± 0.3 ng/mL; P = 0.09), number of follicles > 10 mm (3.7 ± 2 vs. 4.1 ± 2 mm; P = 0.18), and thickness of endometrium (10.9 ± 1.7 vs. 11.0 ± 2.5 mm; P = 0.79). However, patients obtained higher number of oocytes (3.4 ± 1.9 vs. 4.1 ± 2.1; P = 0.03) and received longer stimulation duration (8.7 ± 1.8 vs. 9.6 ± 2.1 days; P = 0.003) in the control group than the early stop group. No difference was found in number of embryos transferred (2.3 ± 1 vs. 2.6 ± 1; P = 0.08). The clinical characteristics of the patient cohort are showed below (Table 2).

**Table 2:** IVF characteristics of the two groups with early stop of progesterone or continuation.

	Early stop group (N = 100)		Control group (N = 56)		P-value
Protocols					0.15 ^NS^
GnRH-antagonist	72	(72.0%)	34	(60.7%)	
GnRH-agonist	28	(28.0%)	22	(39.3%)	
Baseline FSH level (mIU/mL)	12.8 ± 3.8	(2.4–24.3)	13.7 ± 6.4	(5.14–36.8)	0.36 ^NS^
Baseline LH level (mIU/mL)	5.0 ± 1.8	(0.8–9.2)	5.7 ± 4.3	(0.9–22.5)	0.23 ^NS^
Baseline estradiol level (pg/mL)	36.2 ± 17.6	(20–131)	34.8 ± 13.8	(0.3–84)	0.60 ^NS^
Stimulation duration (days)	8.7 ± 1.8	(3–13)	9.6 ± 2.1	(6–14)	0.003*
Estradiol on hCG triggering day (pg/mL)	797.7 ± 475.2	(87–2598)	953.5 ± 530.8	(155–2523)	0.06 ^NS^
P on hCG triggering day (ng/mL)	0.5 ± 0.6	(0.2–5.7)	0.6 ± 0.3	(0.2–2.17)	0.09 ^NS^
Number of follicles >10 (mm)	3.6 ± 1.8	(1–7)	4.0 ± 1.8	(1–7)	0.15 ^NS^
EM thickness (mm)	10.9 ± 1.7	(6.6–15)	11.0 ± 2.5	(7.0–18.1)	0.79 ^NS^
Number of oocytes retrieved	3.4 ± 1.8	(1–8)	4.1 ± 2.1	(1–8)	0.03*
Number of embryos transferred	2.3 ± 1.0	(1–4)	2.6 ± 1.0	(1–4)	0.08 ^NS^

Data are means ± SD for continuous variables and number (%) for categorical variables

^NS^: not statistically significant

*: statistically significant

P: progesterone

EM: endometrium.

No significant difference was observed in progesterone level (64.5 ± 53.3 vs. 65.3 ± 43.3 ng/mL; P = 0.92) and hCG level (402.0 ± 380.4 vs. 432.2 ± 394.9 ng/mL; P = 0.64) on the day of pregnancy test (post-OPU 16 days). A similar number of gestational sac (0.9 ± 0.6 vs. 0.9 ± 0.7 ng/mL; P = 0.79) was found in the two groups. No significant difference was noted in biochemical pregnancy (19.0% vs. 26.8%; OR = 0.64; P = 0.26), implantation rate (40.2% vs. 34.5%; OR = 1.28; P = 0.27), clinical pregnancy rate (55.0% vs. 57.1%; OR = 0.92; P = 0.80), ongoing pregnancy rate (47.0% vs. 46.4%; OR = 1.02; P = 0.95), miscarriage rate (34.0% vs. 26.7%; OR = 1.41; P = 0.35) and live-birth rate (44.0% vs. 46.4%; OR = 0.91; P = 0.77) between the early stop of progesterone supplement and continuation groups, respectively. A similar percentage in multiple pregnancies was found in the two groups. The pregnancy outcomes are described below (Table 3).

**Table 3:** Pregnancy outcomes for the early stop of progesterone supplement and control groups.

	Early stop group (N = 100)		Control group (N = 56)		OR (95% CI)		P-value
P level at post-OPU 16 days	64.5 ± 53.3	(2.0–343)	65.3 ± 43.3	(3.5–238)			0.92 ^NS^
hCG level at post-OPU 16 days	402.0 ± 380.4	(20.4–2073)	432.2 ± 394.9	(23.6–1842)			0.64 ^NS^
Number of gestational sac	0.9 ± 0.6	(0–3)	0.9 ± 0.7	(0–3)			0.79 ^NS^
Implantation rate (%)	92/229	(40.2%)	50/145	(34.5%)	1.28	(0.83–1.97)	0.27 ^NS^
Biochemical pregnancy rate (%)	19	(19.0%)	15	(26.8%)	0.64	(0.30–1.39)	0.26 ^NS^
Clinical pregnancy rate (%)	55	(55.0%)	32	(57.1%)	0.92	(0.47–1.77)	0.80 ^NS^
Ongoing pregnancy rate (%)	47	(47.0%)	26	(46.4%)	1.02	(0.53–1.97)	0.95 ^NS^
Miscarriage rate (%)	34	(34%)	15	(26.7%)	1.41	(0.68–2.90)	0.35 ^NS^
Live-birth rate (%)	44	(44.0%)	26	(46.4%)	0.91	(0.47–1.75)	0.77 ^NS^
Singleton	39/44	(88.6%)	19/26	(73.1%)			
Twins	5/44	(11.4%)	6/26	(23.1%)			
Triplets	0/44	(0%)	1/26	(3.8%)			

Data are number (%) for categorical variables. OR = Odds ratio, with 95% confidence interval (CI).

P: progesterone; OPU: ovum pick-up

^NS^: not statistically significant.

Multivariate logistic regression analysis was performed when stimulation duration, number of oocytes retrieved were adjusted. The analysis with adjusted odds ratios (AORs) revealed no significant difference in clinical pregnancy rate (AOR = 1.72, 95% CI: 0.35–1.45, P = 0.35), ongoing pregnancy rate (AOR = 0.86, 95% CI: 0.43–1.71, P = 0.66), miscarriage rate (AOR = 1.06, 95% CI: 0.89–1.26, P = 0.66) and live-birth rate (AOR = 0.75; 95% CI: 0.37–1.50, P = 0.41) between the early stop of progesterone supplementation and continuation groups. Overall reproductive outcomes in multivariate logistic regression analysis are described below (Table 4).

**Table 4:** Overall reproductive outcomes in multivariate analysis.

	AOR (95% CI)		P value
Clinical pregnancy rate	1.72	(0.35–1.45)	0.35 ^NS^
Ongoing pregnancy rate	0.86	(0.43–1.71)	0.66 ^NS^
Miscarriage rate	1.06	(0.89–1.26)	0.66 ^NS^
Live-birth rate	0.75	(0.37–1.50)	0.41 ^NS^

Data are number (%) for categorical variables. AOR = adjusted odds ratio, with 95% confidence interval (CI).

^NS^: not statistically significant.

## Discussion

In this study, we first demonstrate that we can safely stop progesterone supplementation right after confirmation of positive pregnancy test for patients of POR in fresh ET cycles using hCG for LPS. There are no significant differences of pregnancy outcomes regarding clinical pregnancies, ongoing pregnancies, miscarriage and live births between early stop group and control group. The 1500 IU hCG was given three times on day 2, 5 and 8 after OPU during the luteal phase that is sufficient to support corpus luteal function [29]. After implantation, hCG produced by embryo entering the maternal circulation sustain the CL [3]. CL will continue secretion of estrogen and progesterone. Then the placenta will gradually take over the function of CL and secrete sufficient steroid hormones to maintain the pregnancy. The prolonged supplement of progesterone is not necessary in this situation.

Several randomized controlled trials found no statistically significant differences in miscarriage, ongoing pregnancy and live birth rates between IVF patients who underwent early progesterone cessation and those who received progesterone continuation to 10 to 12 weeks [7,17,18]. A recent meta-analysis concluded that the currently accessible evidence suggests that progesterone use beyond the first positive hCG test might generally be unnecessary [30]. However, large-scale randomized controlled trials are needed to reinforce this conclusion. So far, clear evidence regarding when to discontinue progesterone is not available. Some investigators suggested that an early stop can be based on assessing endogenous CL activity by detection of serum progesterone and estradiol levels on the day of pregnancy test [31,32]. The use of hCG for LPS that maintain the function of CL, and the progesterone serum level is high at confirmation of pregnancy. It is considered reasonable to stop progesterone supplementation.

Progesterone, estrogen, hCG or GnRH agonist have been used for LPS in IVF/ICSI-ET cycles, although the ideal method has not been obviously determined [9–15]. The hCG has been cautioned for its increasing risk of OHSS. A previous study revealed that at women with ≥ 13 follicles on the day of hCG were at increased risk of developing moderate to severe OHSS, while women with ≥ 18 follicles were at increased risk of severe OHSS [33]. Independent risk factors associated with the development of OHSS have been identified as low basal FSH level, high peak estradiol after ovarian stimulation and a high number of growing follicles [34,35]. In our study, all patients included were all poor responders. They have relative high basal FSH level, low peak estradiol after stimulation and low number of growing follicles. The risk of OHSS is very low, and there were no any moderate or severe OHSS in this study. The hCG for LPS is reasonable and safe for poor ovarian responders. However, the hCG should not be used in the normal or high ovarian responders for LPS.

The general attitude among most practitioners has been that it is better to continue progesterone than to take a risk of a pregnancy loss using the earlier stop [19–21]. However, assisted reproductive technology (ART) treatment for infertility couple is not an easy task. Progesterone supplementation is considered to be safe and harmless but is a source of complaints by patients. Those complaints mainly originate from messy vaginal leakage with swelling and itching vulva for patients receiving vaginal progesterone suppository; headache and dizziness for patients receiving oral progesterone pills; or painful sensation or abscess formation for patients receiving intramuscular injections of progesterone in oil. Therefore, we suggest patients of POR using hCG for LPS that progesterone can be stopped after confirmation of pregnancy. This strategy can decrease adverse effects of extended progesterone supplementation and reduce the total treatment burden and cost.

There are some limitations of our study that should be considered, including the retrospective nature of the analysis and the somewhat small sample sizes. Patients who were pregnant with serum level hCG < 20 IU/L and lost follow-up were excluded in the study. It is possible to cause selection bias. The early stop or continuation of progesterone supplementation was dependent on different doctor’s ideas. This decision can be treated as a random process. Therefore, the similar outcomes between the early stop group and control group were not the results of the doctors’ clinical judgment. Three doses of hCG for LPS may be sufficient without addition of progesterone supplementation during the same period. We need more research to prove this point of view in the future. For the patients of POR using progesterone only, without hCG, for LPS, whether the early stop of progesterone can be applied to every patient or not is not included in the scope of this paper. The degree of CL rescue may depend on interaction of multiple variables including response of ovarian stimulation, dynamics clearance of hCG which used for trigger, degree of pituitary suppression during luteal phase by GnRH agonist or antagonist and timing of endogenous hCG produced by the newly formed placenta. This condition may deserve further scrutiny in future research.

This study is based on the Chinese in Taiwan. Before drawing definite conclusions, it is needed to be careful because the results might not be extendible to the people in the other countries/regions. For instance, in some countries/regions, the pregnant women might change diets habits during pregnancy which might serves similar purpose as progesterone.

## Conclusions

The results of this study indicate that progesterone supplementation can be safely discontinued on the day of positive pregnancy test for patients of POR using hCG as LPS in fresh ET cycles. There was no difference in implantation, clinical pregnancy, ongoing pregnancy, miscarriage, and live birth rates with or without early stop of LPS. There were no moderate or severe OHSS in poor responder using hCG for LPS. It is important because this strategy will allow women with POR to stop their progesterone supplementation several weeks early, leading to cost-savings and reduction of side effects and discomfort of progesterone use.

## Supporting information

S1 File Dataset. (XLSX)
